# Tumor Genome Wide DNA Alterations Assessed by Array CGH in Patients with Poor and Excellent Survival Following Operation for Colorectal Cancer

**Published:** 2007-10-12

**Authors:** Kristina K. Lagerstedt, Johan Staaf, Göran Jönsson, Elisabeth Hansson, Christina Lönnroth, Ulf Kressner, Lars Lindström, Svante Nordgren, Åke Borg, Kent Lundholm

**Affiliations:** Department of Surgery, Surgical Metabolic Research Laboratory at Lundberg Lab. for Cancer Research, Sahlgrenska University Hospital, Göteborg University, SE 413 45 Göteborg, Sweden; 1 Department of Oncology, University Hospital, Lund, Sweden; 2 Department of Surgery, Uddevalla Hospital, SE 451 80 Uddevalla, Sweden

**Keywords:** Colorectal cancer array CGH, Tumor DNA

## Abstract

Genome wide DNA alterations were evaluated by array CGH in addition to RNA expression profiling in colorectal cancer from patients with excellent and poor survival following primary operations.

DNA was used for CGH in BAC and cDNA arrays. Global RNA expression was determined by 44K arrays. DNA and RNA from tumor and normal colon were used from cancer patients grouped according to death, survival or Dukes A, B, C and D tumor stage. Confirmed DNA alterations in all Dukes A – D were judged relevant for carcinogenesis, while changes in Dukes C and D only were regarded relevant for tumor progression.

Copy number gain was more common than loss in tumor tissue (p < 0.01). Major tumor DNA alterations occurred in chromosome 8, 13, 18 and 20, where short survival included gain in 8q and loss in 8p. Copy number gains related to tumor progression were most common on chromosome 7, 8, 19, 20, while corresponding major losses appeared in chromosome 8. Losses at chromosome 18 occurred in all Dukes stages. Normal colon tissue from cancer patients displayed gains in chromosome 19 and 20. Mathematical Vector analysis implied a number of BAC-clones in tumor DNA with genes of potential importance for death or survival.

The genomic variation in colorectal cancer cells is tremendous and emphasizes that BAC array CGH is presently more powerful than available statistical models to discriminate DNA sequence information related to outcome. Present results suggest that a majority of DNA alterations observed in colorectal cancer are secondary to tumor progression. Therefore, it would require an immense work to distinguish primary from secondary DNA alterations behind colorectal cancer.

## Introduction

It is assumed that colorectal cancer development constitutes an evolutionary process and a stepwise accumulation of required genetic alterations leading to increased malignancy (Fearon and Vogelstein, 1990). Around 15% of colorectal tumors are characterized by microsatellite instability (MSI or MIN) in combination with various mutations due to deficient DNA mismatch repair (MMR) genes (Kinzler and Vogelstein, 1996). The majority of malignant colorectal tumors are however characterized by chromosomal instability (CIN) which refers to the appearance of gross chromosomal aberrations including gain and loss of large DNA regions or even whole chromosomes (Lengauer et al. 1998; Rajagopalan et al. 2003). CIN leads to increased inability to maintain genome integrity, although the precise order of genomic events is less defined. Opposite to CIN tumors, MSI neoplasms typically retain a near-diploic karyotype and show near normal frequencies of gross-chromosomal aberrations (Bhattacharyya et al. 1994; Parsons et al. 1993; Eshleman et al. 1998). However, aneuploid changes typical for CIN tumors may occur early in low graded dysplastic adenomas, and are therefore proposed as major factors behind progression of colon cancer (Hermsen et al. 2002), although recent observations have questioned whether genetic instability precedes tumor formation (Cardoso et al. 2007). The development of advanced techniques such as high-resolution microarrays (Pinkel et al. 1998; Pollack et al. 1999; Snijders et al. 2001; Ishkanian et al. 2004) provides possibilities for a variety of detailed genome-wide screening of DNA copy number changes in malignant tumors as well as epipenetic alterations (Pinkel and Albertson, 2005; Cardoso et al. 2007). Seen together appearing results reveal an unexpected magnitude and complexity of genetic damage in both coding and non-coding regions, in various stages of colorectal cancer (Douglas et al. 2004; Nakao et al. 2004; Buffart et al. 2005; Mehta et al. 2005; Jones et al. 2005; Camps et al. 2006). In the present study, we describe quantitative DNA alterations by array CGH analysis in macrodissected colorectal cancer tissue as related to disease stage and survival following primary operations aimed for cure. Our results add to published information particularly on the difference of DNA alterations in tumors from patients with early relapse and death compared to cured patients.

## Materials and Methods

### Patient groups

The patient material comprised 64 patients operated on for sporadic primary colorectal carcinoma. Thirty-two patients who underwent primary surgery in Uppsala county, Sweden between 1988–1990 were subdivided into two groups according to survival. Nineteen patients alive 200 months after primary surgery were grouped as “alive.” Thirteen patients who died because of colorectal cancer within 12 months after their primary operation were grouped as “dead.” Alive patients comprised 6 males and 13 females classified as 4 Dukes A, 11 Dukes B, and 4 Dukes C; 21% had MSI positive tumors and 53% had tumors with p53 mutations. Dead patients comprised 7 males and 6 females classified as 3 Dukes B, 3 Dukes C and 7 Dukes D; 31% had MSI positive tumors and 62% had tumors with p53 mutations as described elsewhere (Lagerstedt et al. 2005).

Additional 32 patients were included following primary operations in Uddevalla County of Sweden between 2001–2003 and were grouped according to tumor stage by the Dukes A–D classification. Each category of Dukes A, B, C and D comprised 8 patients with 4 males and 4 females, except the Dukes D group, which contained 5 males and 3 females. None of the 64 patients underwent any additional treatment beside surgery according to our institutional standard procedures at the time of operation.

### BAC array construction and procedures

Microarrays with complete genome coverage were produced from the 32K BAC clone library (CHORI BACPAC Resources, http://bacpac.chori.org/genomicRearrays.php) by the Swegene DNA Microarray Resource Center, Department of Oncology, Lund University, Sweden (http://swegene.onk.lu.se). DOP-PCR products were obtained from BAC DNA template and purified using filter based 96-wells (PALL), dried and re-suspended in 50% DMSO. Arrays were printed on UltraGAPS slides (Corning) using a MicroGrid II spotter (Biorobotics) as described in details elsewhere (Jonsson et al. 2005a). BAC clones were mapped according to the Human May 2004 Genome freeze (UCSC Genome Bioinformatics, http://www.genome.ucsc.edu).

Six 32K tiling BAC arrays were used to determine DNA copy number alterations in pooled tumor DNA from patients grouped as dead, alive, Dukes A, B, C and D in comparison to reference DNA (Human Genomic DNA from whole blood, Clontech, BD Biosciences). Array was run on tumor DNA from dead patients versus tumor DNA from alive patients. Normal colon tissue DNA from dead and alive patients was also hybridized against reference DNA. cDNA array analyses of DNA were also used to compare with observations found in BAC array analyses (Fig. 4).

Overall chromosomal aberrations were given as the number of BAC clones considered altered (gain or loss of copy number) divided by the total number of clones in the genome wide evaluation where X and Y-chromosomes were excluded.

DNA was extracted from fresh frozen primary colorectal carcinomas and normal colon tissue (down to serosa layer) with Qiamp DNA Mini kit (Qiagen) according to instructions. All tumors contained around 60–80% neoplastic cells according to separate estimates, with remaining 20%–40% containing endothelial, stromal and inflammatory cells. Sample labeling and hybridization were performed as described (Jonsson et al. 2005a). Briefly, 1.5–3 μg genomic DNA from patients and reference DNA was differentially labeled with Cy5-dCTP or with Cy3-dCTP (Amersham Biosciences) using random primer labeling (Bioprime array CGH genomic Labeling module, Invitrogen). Labeled sample and reference DNA were mixed and unincorporated nucleotides were removed using CYScribe GFX purification kit (Amersham Biosciences) prior to coprecipitation with human Cot-1 DNA. The labeling reactions were applied to arrays and incubated for 72 h at 37 °C. Slides were washed and scanned in Agilent microarray scanner (Agilent Technologies). Identification of individual spots on scanned arrays was performed with GenePix Pro 4.0 (Axon Instruments).

### cDNA array construction and procedures

cDNA microarrays containing 27,648 sequence-verified IMAGE clones from the Research Genetics IMAGE clone library were obtained from the Swegene DNA Microarray Resource Center at Lund University (http://swegene.onk.lu.se). 6 μg of sample and reference DNA were labeled and hybridized according to previously described procedures for BAC arrays except that cDNA arrays were hybridized at 42 °C.

### RNA extraction and microarray expression

Tumor and normal colon tissue RNA was either extracted with TRIzol reagent (Invitrogen Life Technologies). mRNA was linearly amplified with BD smart mRNA amplification kit (BD Biosciences, Clontech, Palo Alto, CA, U.S.A.), or extracted with Rneasy Fibrous Tissue Kit (Qiagen) where mRNA was selected with mRNA Purification Kit (Amersham Biosciences). RNA fractions were quality controlled in a Bioanalyzer (Agilent Technologies) and quantified by a NanoDrop ND-1000A Spectrophotometer (NanoDrop Technologies Inc). 400 ng polyA + mRNA from tumor and normal colon were labeled with Cy3-dCTP and Cy5-dCTP respectively (Amersham Biosciences) with Agilent Fluorescent Direct Label Kit and samples were hybridized to 44K Human Whole Genome Oligo Microarrays (Agilent Technologies) using the In situ Hybridization Kit Plus (Agilent Technologies), incubated at 60 °C for 18 hours and scanned on an Agilent Microarray scanner. Three patients were hybridized individually (with technical replicates, dye-swaps) and six patients were pooled and run as a single experiment. Data were processed in Feature Extraction Software, v.7.5 (FE) (Agilent Technologies), background was subtracted, outliers flagged and dyes were normalized with linear and lowess. Processed signals from FE output files were imported into GeneSpring Software, v.7.2 (Silicon Genetics, Agilent Technologies) with Agilent Feature Extraction plug-in. Dye-transformation of specified samples, normalizations per spot/divided by control channel as well as per chip/normalized to 50th percentile and filtering on flags were performed. Processed data from three individual patients and a pool of six patients were combined and the 99% confidence interval was calculated from merged data to identify genes with aberrant expression. Patient data represent gene expression in tumors from Dukes A (1), Dukes B (2), Dukes C (4) and Dukes D (2) from five females and four males.

### DNA image analysis, data processing and statistics

Images were quantified on an Agilent G2565AA microarray scanner (Agilent Technologies, Palo Alto, CA). Fluorescence intensities were extracted using the Genepix Pro 4.0 software (Axon Instruments Inc, Foster City, CA) uploaded into Bio Array Software Environment (BASE) open source software (http://base.thep.lu.se) for further analysis (Saal et al. 2002). Data analysis was performed in BASE as described (Jonsson et al. 2005b). Briefly, intensity ratios for each spot were obtained by calculating background corrected Cy3 and Cy5 intensities from the median and local background pixels. Spots with Cy3 and Cy5 intensities >65000 and a signal to noise ratio <1.5 and a spot radius >40 were excluded from the data set in BAC analyses, while cDNA ratios in spots were handled similarly without any restriction in signal intensities. Spots indicated as flags by the Genepix software were removed prior to normalization by the Lowess curve fit method for both platforms (Yang et al. 2002). A moving average of three clones was applied and BASE implementation of CGH Plotter was used to determine deletion/amplicon boundaries (Autio et al. 2003). Noise constant was set to 15 and amplification/deletion limits was set to log(2) values of ±0.2. High reproducibility considering log(2) values was obtained for all BAC clones within the 32K array with a mean SD of 0.135 in self versus self hybridizations. Further, analysis of cells with different numbers of X-chromosomes, demonstrated a linear rise in log(2) values for X-clones (unpublished). Mapping information was retrieved from the USCS Genome Browser (March 2005 freeze). The uniformity of log(2) ratio distribution in chromosomes as well as complete data sets were tested and confirmed by the Kolmogorov-Smirnoff test. Only autosomal clones were included in the analysis. The SD calculated from log(2) ratios from all samples was 0.14. Differences between samples were analyzed with χ^2^-test and corrected by Bonferroni statistical adjustments.

Vector analysis was performed on data from hybridization of tumor DNA from dead and alive patients vs reference DNA from normal subjects. Net alterations in hybridization log(2) ratios were graphed in a two-dimensional coordinate system, where the different quadrants confirm conditions or events that promote death or alive events directly or indirectly related to genetic deviations compared to normal reference DNA.

## Results

### Genome wide alterations in tumor tissue vs normal colon tissue

The number of aberrant clones ranged from 1–15% (genome wide) to 82% for individual chromosomes in tumor DNA (Table 1). Copy number gains were significantly more common than loss of DNA sequences (p < 0.01). Structural DNA alterations in tumor tissue versus normal DNA were found in each chromosome. Chromosomes with the highest prevalence of altered BAC clones were 8, 13, 18 and 20 and least altered chromosomes were 1, 2, 3, 5, 6 and 11. The size of copy number loss ranged from 4 to 351 BAC clones corresponding to 210 kbp to 36 Mbp. The extent of gains and amplifications ranged from 2 to 599 BAC clones, corresponding to 27 kbp to 55 Mbp. No incidence of homozygous deletions was observed.

**Figure 1.**
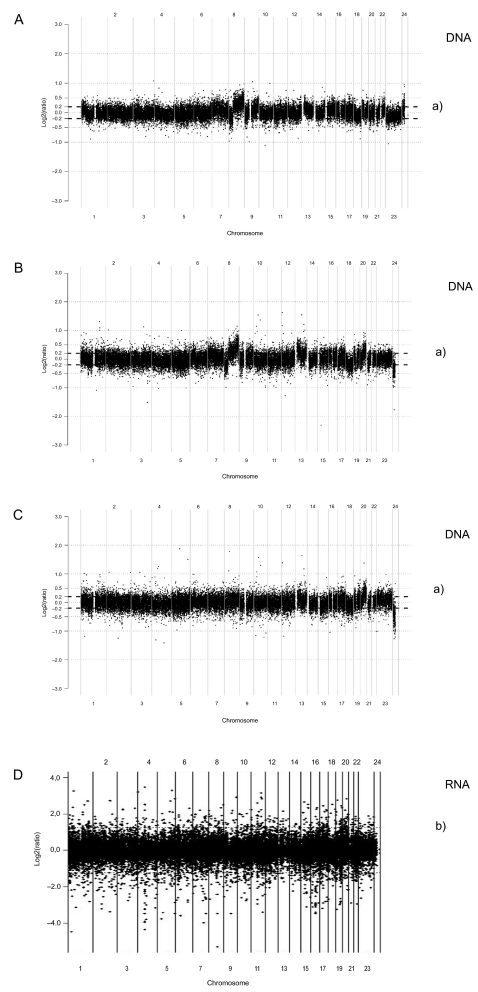
Genome wide array CGH BAC analysis. Tumor DNA from dead patients was hybridized against tumor DNA from alive patients **(A)**; tumor DNA from dead patients vs normal reference DNA **(B)**; tumor DNA from alive patients vs normal reference DNA **(C)**. Relative chromosomal copy number is given on the y-axis as the log(2) ratio. Each ratio represents a BAC clone on the array. Values of log(2) ratios above 0.2 were regarded gain of copy number and log(2)ratios below −0.2 were considered loss of copy number. Alive patients were cured from colorectal cancer with more than 10 years survival, while dead patients did not survive beyond 1 year following their primary operation. a) is the ± 0.2 log(2) ratio (∼95% confidence limit) determined by CGH plotter analysis software. Panel D shows RNA expression in tumor tissue vs normal colon tissue RNA from a comparable group of 9 cancer patients (Dukes A – D) selected by chance from the main patient cohort. b) represents ±2.6SD (99% confidence interval).

RNA expression profiles in tumor tissue from colorectal cancer patients of the same cohort displayed 78 genes with significantly increased expression and 140 genes with decreased expression in tumor tissue vs normal colon tissue. Figure. 1D shows the spectrum of expression along the genome compared to observed structural DNA alterations (Figs. 1A–C).

### Genome wide DNA alterations in tumor tissue from dead and alive patients

**Figure 2.**
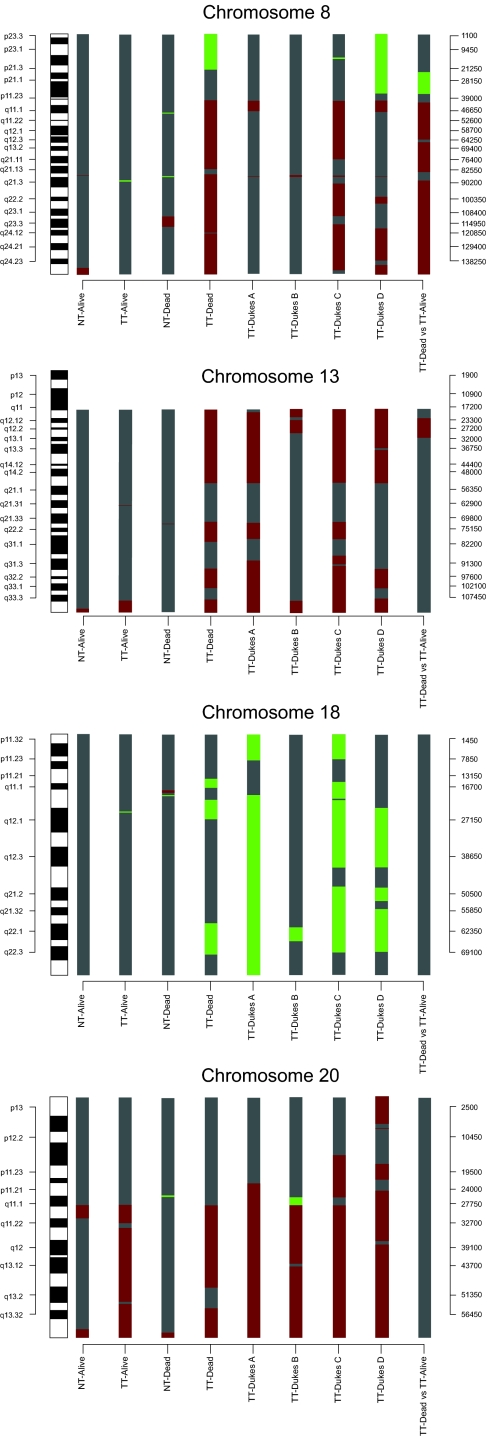
Results from 9 array based CGH analyses on chromosome 8, 13, 18 and 20 in tumor DNA (TT) and normal colon tissue DNA (NT). Green bars represent loss of copy number and red represents gain of copy number. Gray is DNA sequences without statistically significantly structural genomic alterations.

**Figure 4.**
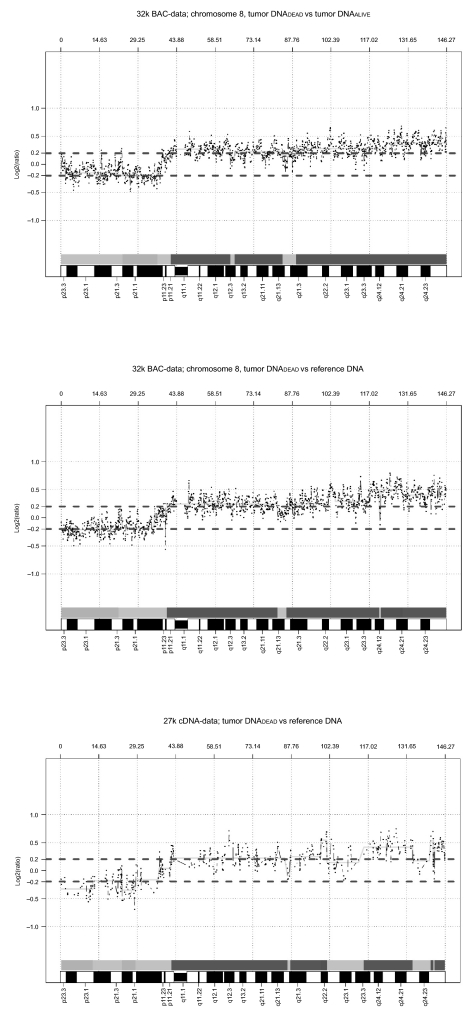
Copy number gain and loss in chromosome 8 based on three CGH arrays where tumor DNA from dead patients was hybridized either to DNA from alive patients or to reference DNA. (A: 32k BAC array; B: 27k cDNA array; C: 32k BAC array). The statistical confidence interval was ±0.2 log(2) ratio.

Four percent, 8% and 2% of the BAC-clones of autosomal chromosomes were altered in tumor DNA analyzed from various sets of hybridization; (dead/alive, dead/reference, alive/reference) (Table 1). Copy number gain was more common than loss (p < 0.01) and dead patients had a higher frequency of genome wide gain and loss in tumor DNA than alive patients (p < 0.01). Several chromosomes showed major DNA alterations, namely chromosomes 8, 13, 18 and 20 in tumor tissue (Table 1, Fig. 2).

### Copy number changes in tumor DNA from dead vs alive patients

#### Tumor DNA vs tumor DNA

Gain was found for 63% of the clones covering chromosome 8 starting at the 8p-arm (6.3 Mb at 8p11.21–8p11.1) and covering 92% of the q-arm (Table 2). A 17 Mb region on chromosome 9 (9q33.2–9q34.3) and a 9 Mb region on chromosome 13 (13q12.12–13q13.1) were gained, which represented 15% and 8% of all clones covering respective chromosome (Fig. 1, Table 2). A major loss was observed at 8p21.3–8p12 (Table 2).

#### Tumor DNA vs reference DNA

Sixty-seven percent of the clones covering chromosome 8 were gained in tumor DNA from dead patients with start at the 8p-arm (6.3 Mb at 8p11.21–11.1) covering 97% of the q-arm (Fig. 2). Likewise, 61% of BAC-clones in chromosome 13 were gained in tumor DNA from dead patients. Three restricted regions were found at 13q11–13q14.2 (35 Mb), 13q21.33–13q31.1 (9 Mb) and 13q31.3–13q33.1 (9 Mb) (Fig. 2). Loss of DNA at 8p occurred in tumor DNA from dead patients, where a 22 Mb deletion started at 8p23.3–8p21.3. Three regions with copy number loss in chromosome 18 were found in dead patients corresponding to 25% of the BAC-clones (3 Mb starting at 18p11.21, 6 Mb at 18q11.1–18q12.1 and 9 Mb starting at 18q21.33–18q22.3) (Fig. 2).

### DNA alterations related to tumor progression (Dukes A + B, C + D)

Seven percent, 4%, 15% and 12% of BAC-clones representing autosomal chromosomes were altered in Dukes A, B, C and D tumors respectively (Table 1). Copy number gain was significantly more common than copy number loss in all Dukes stages (p < 0.01). Gains were found in chromosome 7 (9%–45%), 8 (1%–55%) and 20 (46%–76%) in Dukes A–D (Table 2). Copy number loss was observed in 6%–61% of all BAC-clones representing chromosome 18 in all Dukes classes (Fig. 2).

#### Copy number changes in Dukes A + B and Dukes C + D

Tumor DNA from Dukes C + D displayed additional gains in chromosome 7, 8, 19 and 20 compared to Dukes A + B. Major loss of DNA sequences in chromosome 18 was similar between Dukes A + B and C + D while chromosome 8 showed major loss in Dukes C + D not observed in Dukes A + B (Table 2). Candidate genes related to tumor progression in control of cell proliferation and apoptosis are shown in Table 4 and 5.

Normal colon tissue DNA from cancer patients displayed gain in chromosome 19 and 20 compared to normal reference DNA (Table 2).

### Large-scale copy number variation in normal colon tissue DNA

Confirmed and unconfirmed large-scale copy number variaton was observed in normal colon tissue from cancer patients with different clinical outcome (Iafrate et al. 2004; Sebat et al. 2004; Eichler, 2006). These changes are summarized in Table 3. Alive patients displayed only confirmed CNV locus while both confirmed and unconfirmed DNA alterations occurred in our dead patients. Such unconfirmed DNA locus were evaluated for candidate genes with importance for tumor progression according to proliferation or apoptosis (Table 6).

### Vector analysis

**Figure 5.**
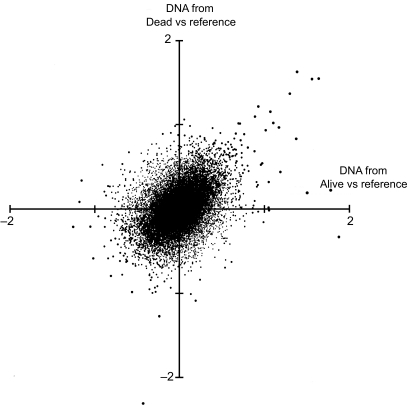
Distributions of log(2) in BAC-clone hybridizations of tumor DNA from dead and alive patients versus normal reference DNA as described in Methods. The upper left and lower right quadrants represent conditions where both non-surviving and surviving patients have pronounced and less pronounced DNA alterations compared to normal reference DNA. Thus, in the upper left quadrant the non-surviving patients have pronounced and surviving patients less pronounced DNA alterations compared to normal individuals. The lower right quadrant represents opposite conditions.

Figure 5 demonstrates distributions of DNA alterations between dead and alive patients in whole genome hybridizations versus normal reference DNA. Each observation indicates its proportional weight in vectors moving either towards death or survival. According to this plot, we ranked the 20 most extreme BAC-clones contributing to death events due to copy number gain or loss. Genes in these DNA regions represent candidates related to disease specific mortality as presented in Table 7.

## Discussion

The present study evaluates structural (sequence) alterations in DNA isolated from tumor tissue obtained at primary curative resections of colorectal cancer. Several earlier studies have addressed relationships between DNA aberrations and disease progression, outcome and response to adjuvant treatment (De Angelis et al. 2001; Bardi et al. 2004; Diep et al. 2004; Chang et al. 2006; Diep et al. 2006). The traditional approach in such efforts is to investigate a number of patients with statistical power to relate genetic alteration to survival and treatment response. This approach, with genome wide analyses on material from individual patients on large cohorts, is restricted by financial costs and statistical aspects in microarray analyses. Therefore, we chose an alternative approach with analyses on pooled DNA prepared from individual patients grouped according to clinical outcome or tumor stage (Dukes A–D), which represents a more robust model with less by chance variation considering the large number of clones (∼32000) in each assay. Thus, a model based on pooled DNA and RNA provides more stabilized information by canceling out random variations as emphasized by Cardoso et al. (Cardoso et al. 2007). Patients with either poor or excellent survival following surgery were selected from a large cohort of patients with colorectal cancer selected by chance and subjected to standard treatment at our institution. In a group from all operated patients during 1990 and 2002 we randomly selected 13 patients who died in colorectal cancer within 12 months vs 19 patients who survived for more than ten years, which is statistically equal to be cured. A limitation in analyses on pooled DNA is that small but significant structural alterations may be unidentified and thereby decrease the sensitivity of analyses. However, as a screening procedure for evaluation of major factors, our approach is statistically superior. In order to decrease the risk for misinterpretations in conclusion of results from dead vs alive patients we also confirmed such results by hybridization of tumor DNA vs reference leucocyte DNA commercially available from healthy individuals. Given the existence of copy number variants of relatively high frequency in general population (Redon et al. 2006) it may not be beneficial to analyze a defined “normal reference DNA.” However, this comparison is regarded on internal analytical standard being commercially available world wide.

DNA alterations detected in the surgically removed tumors represents the sum of changes accumulated during disease progression (Rajagopalan et al. 2003; Michor et al. 2005). It is possible that certain alterations are critical for carcinogenesis while other may promote invasive growth and metastatic spread (Buffart et al. 2005; Mehta et al. 2005; Ghadimi et al. 2003; Saha et al. 2001). Considerable efforts have been devoted to delineate differences between early and late events in colorectal cancer development. (Lengauer et al. 1998; Lengauer et al. 1997). Theoretically, it may well be that critical genetic events during carcinogenesis are less important for tumor progression and vise versa (Hunter, 2004). Here, we approached this concept by comparing DNA alterations in patients with tumors of well-defined clinical stage according to Dukes. Accordingly, patients with tumor stage of Dukes A and B have world wide clear cut better outcome compared to patients with Dukes C and D stage. Therefore, when a defined DNA alteration occurs in all Dukes A–D stages, and is not present in normal tissue, it should be related to carcinogenesis and early progression. On the other hand, when alterations appear in Dukes C and D tumors only, they should be associated with tumor progression.

In general, our study reveals that DNA copy number gains are more frequent than losses in colorectal cancer. Based on above principles we observed a number of alterations that distinguish tumors with excellent versus poor prognosis, most obvious being the alterations on chromosome 8. Loss of a limited region on 8p (8p23–8p21) occurred more often in patients of poor prognosis, whereas gain of a large proportion of chromosome 8 (including most of 8q) characterized these tumors. Frequent DNA copy number gain at 9q33.2–9q34.3 and 13q12.12–13q13.1 in tumors of poor prognosis may reveal the existence of activated oncogenes that confer aggressive disease. These tumors also contained frequent losses at three limited regions on chromosome 18, including two 18q regions that suggest the existence of important tumor suppressor genes. However, copy number loss on chromosome 18 was seen in both Dukes A + B and C + D tumors, suggesting that these are early events in colorectal tumor development. Contrary, Dukes C + D tumors displayed gains on chromosome 7, 8, 19 and 20 in comparison with Dukes A + B tumors, supposedly related to tumor progression. Losses on chromosome 8p where observed in Dukes C + D but not in Dukes A + B. Unexpectedly, it was observed that normal colon tissue harbored quantitative DNA alterations (gains at chromosome 19 and 20) also found in Dukes C + D tumors, which contradict their connection to tumor progression. These DNA alterations may reflect the toxic environment that colon epithelial is exposed to during life-time predisposing to carcinogenesis, but it may also represent CNVs among different subject populations.

Several studies have implied critical DNA alterations that predict clinical outcome. Many such reports have been evaluated in less complex experimental models as cultured tumor cells, where signal transduction pathways in control of cell proliferation and apoptosis are well described. However, overall genomic aberrations observed in the present material appear a major challenge to distinguish primary from secondary DNA alterations. The regions defined here (e.g. on chromosome 8) include several hundred of altered genes that may co-variate with other disease specific alterations without having any primary cause-effect relationship on either carcinogenesis or progression. A hint to this perspective may be to compare structural DNA alterations to significant altered RNA expression along the genome which provide information on DNA alterations in expressed genes (Pollack et al. 2002). Accordingly, Pollack et al. estimated that approximately 12% of variations in gene expression in breast cancer could be attributed to underlying copy number changes. Corresponding rough estimates on the present material may be around 5% considering significantly altered expression versus copy number changes in tumors. Definite information on altered expression versus copy number changes must await analyses on the RNA and DNA from the same tissue specimen (Cardoso et al. 2007), which is under way in our laboratory. Therefore, we used two dimensional vector analysis to sort out the 20 most extreme alterations related to poor survival and found that a majority of these genomic regions (represented by BAC clones) contained only a few known genes that may be related to cancer progression. This dilemma would require more thorough comparisons with gene expression and functional studies and it is not resolved simply by adapting available models of bioinformatics on genomic data. Obviously, there is no simple solution to rank positive and negative factors in prediction of clinical outcome, since it will demand genome-wide analyses on several ten-thousands of patients to resolve such predictions by classic statistics. The situation appears even more problematic considering redundant metabolic pathways to overcome established defects in the control of gene expression including epigenetic changes and micro RNAs (Feinberg et al. 2006; Michael et al. 2003). In this perspective it presently appears an impossible mission to resolve these questions by available models.

In conclusion, the results in the present study demonstrate that tiling array CGH is a powerful approach for genome-wide identification of DNA copy number alterations in pooled DNA from cancer patients. We used pools of tumors from clinically and/or pathologically well-defined patient subgroups selected randomly to sort out only major genomic patterns related to carcinogenesis, tumor progression and prognosis. Despite this approach our results demonstrate an enormous number of DNA sequences that may explain carcinogenesis, tumor growth progression and disease specific mortality. A next step should be to distinguish primary DNA events from secondary covariates to explain disease progression, although this presently seems an overwhelming task.

## Figures and Tables

**Figure 3.**
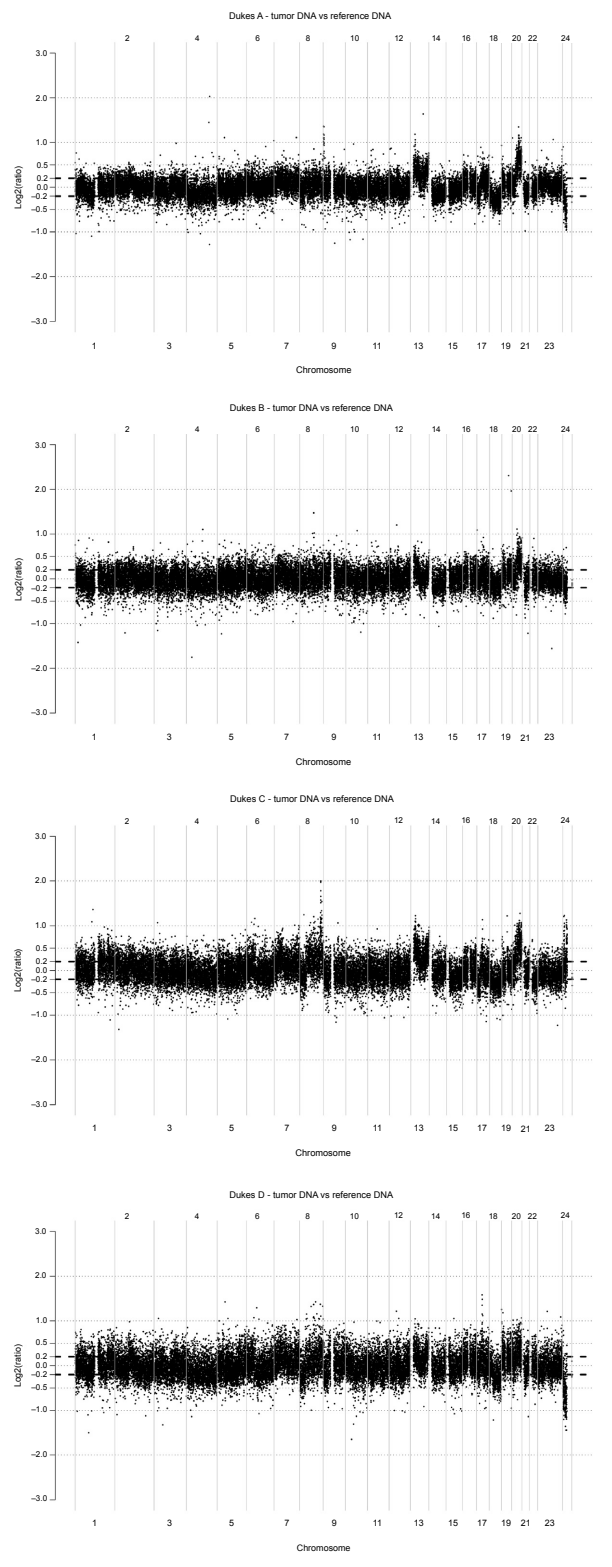
Genome wide array CGH analyses where tumor DNA from patients with colorectal cancer staged as Dukes A, B, C and D respectively was hybridized vs normal reference DNA as described in Methods.

**Table 1.** DNA alterations in tumor tissue and normal colon mucosa adjacent from tumors in patients with colorectal carcinoma grouped according to survival and Dukes stage.

	**Dead vs Alive**	**Dead**	**Alive**	**Dead**	**Alive**	**Dukes A**	**Dukes B**	**Dukes C**	**Dukes D**
				
**Sample**	Tumor DNA vs Tumor DNA	Tumor DNA vs reference DNA		Colon DNA vs reference DNA		Tumor DNA vs reference DNA			
**Chrom**	Altered BAC clones per chromosome (%)^a^								

1	-	2	-	-	-	-	-	14	-
2	-	-	-	≤0.5	-	-	-	2	-
3	-	≤0.5	-	≤0.5	-	-	2	-	-
4	-	≤0.5	1	2	2	8	3	-	1
5	-	-	2	1	-	-	-	≤0.5	-
6	-	-	-	2	-	-	1	5	1
7	≤0.5	1	1	1	-	9.2	-	30	45
8	72	82	≤0.5	5	3	2	1	55	47
9	15	-	2	1	≤0.5	2	16	2	4
10	-	≤0.5	1	2	-	1	2	8	5
11	-	-	-	≤0.5	-	-	-	1	1
12	-	≤0.5	1	≤0.5	2	-	-	2	-
13	8	61	2	≤0.5	2	67	14	70	50
14	≤0.5	-	-	1	-	-	6	1	2
15	-	≤0.5	-	≤0.5	4	-	1	11	4
16	-	≤0.5	2	-	2	5	-	8	9
17	-	-	1	1	44	10	-	28	13
18	-	25	1	1	-	61	6	70	35
19	-	2	8	3	38	2	2	2	32
20	-	45	53	3	7	46	57	73	76
21	-	5	-	2	6	5	16	2	15
22	2	-	1	3	9	-	4	52	4

Genome wide alterations	4.4	8.0	2.1	1.1	3.2	6.8	3.6	14.7	11.6

a Number of altered BAC clones in percent per chromosome.

**Table 2.** Major structural alterations in tumor DNA and normal mucosa DNA related to survival and tumor progression defined by Dukes staging of colorectal cancer.

		**Chromosome**						
**Hybridization**		**7**	**8**	**9**	**13**	**18**	**19**	**20**
**Tumor DNA vs Tumor DNA**								
Dead vs Alive								
	Gain	-	8p11.21 (6 Mb)	9q33.2 (17 Mb)	13q12.2 (9 Mb)	-	-	-
			8q11.21 (16 Mb)					
			8q12.3 (18 Mb)					
			8q21.3 (34 Mb)					
			8q24.13 (22 Mb)					
	Loss	-	8p21.3 (13 Mb)	-	-	-	-	-
**Tumor Progression**								
Dukes A + B								
	Gain	7q36.1 (11 Mb)	8p11.21 (6 Mb)	-	13q12.11 (33 Mb)	-	19p13.3 (2 Mb)	20q11.1 (34 Mb)
					13q21.33 (8 Mb)			
					13q31.3 (22 Mb)			
	Loss	-	-	-	-	18p11.32 (8 Mb)	-	-
						18q11.2 (21 Mb)		
						18q21.2 (21 Mb)		
Dukes C + D								
	Gain	7p22.3 (5 Mb)	8p11.21 (6 Mb)	-	13q12.11 (33 Mb)	-	19p13.3 (2 Mb)	20p13 (7 Mb)
		7p15.3 (33 Mb)	8q22.1 (4 Mb)		13q21.33 (8 Mb)		19p13.3 (17 Mb)	20p12.1 (7.4 Mb)
		7q36.1 (11 Mb)	8q23.3 (28 Mb)		13q31.3 (22 Mb)			20q11.1 (34 Mb)
	Loss	-	8p23.3 (36 Mb)	-	-	18p11.32 (8 Mb)	-	-
						18q11.2 (21 Mb)		
						18q21.2 (21 Mb)		
**Normal mucosa DNA vs Reference DNA**								
	Gain	-	-	-	-	-	19q13.3 (2 Mb)	20q11.1 (2 Mb)
								20q13.33 (1 Mb)
	Loss	-	-	-	-	-	-	-

**Table 3.** Large Scale Copy Number Variation in DNA from normal colon mucosa in dead and alive patients at the time of curative operation for colorectal cancer.

Chromosome	**Normal mucosa DNA from dead patients vs reference DNA**		**Normal mucosa DNA from alive patients vs reference DNA**	
	Confirmed CNP locus	Unconfirmed CNP locus	Confirmed CNP locus	Unconfirmed CNP locus
1		-	-	-
2	2p16.1^f^	-	-	-
3	3p23^h^		-	-
4	4p15.32^b^	-	4p13^g^	-
5	5q13^a^	-	-	-
6	6q11.1^a^, 6q27^h^,^i^		-	-
7	7q11.21^a^, 7q11.23^j^		-	-
8	-	-	8q21.1^a^	-
9	-	-	9p24.3^a^	-
10	10q11.23^k^		-	-
11	10q23.1^j^		-	-
12	-	-	-	-
13	-	-	-	-
14	14q11.2^a^,^b^, 14q31.3^h^	14q11.1	-	-
15	15q15.1^d^	-	15q11.2^e^	-
16	-	-	16p11.2^a^	-
17	17p13.2^j^		-	-
18	-	18q11.2	-	-
19	19p13.3^e^	-	-	-
20	20p11.1^a^,20q13.33^c^	-	20q13.33^c^	-
21	-	-	21q22.3^a^	-
22			22q12.2^a^	
	-	-	22q12.3^c^	-

a Iafrate et al, Nat Genet. 2004, 36:949–951;

b Sebat et al, Science 2004, 305:525–528;

c Tuzun et al, Nat Genet. 2005, 37:727–732;

d Sharp et al, Am J Hum Genet. 2005, 77:78–88;

e De Vries et al, Am J Hum Genet. 2005, 77:606–616;

f Mc Carroll et al, Nat Genet. 2006, 38:86–92;

g Hinds et al, Nat Genet. 2006, 38:82–85;

h Redon et al, Nature 2006, 444:444–54;

i Mills et al, Genome Res. 2006, 16:1182–90;

j Wong et al, Am J Hum Genet. 2007, 80:91–104;

k Locke et al 2006, 79:275–90.

**Table 4.** Copy number gain and loss of genes related to cell proliferation and apoptosis in altered tumor DNA regions as defined in Table 2 from patients with short survival (dead patients).

**Copy number Change**	**Cytoband**	**Proliferation**	**Protein function**	**Apoptosis**	**Protein function**
**Gain**	8p11.21	*ANK1*	Involved in cell activation, proliferation		
	8q22.2			*STK3*	Activation presumably allows cells to resist unfavorable conditions
	8q24.21	*V-MYC*	Transcription factor	*V-MYC*	Multifunctional, nuclear phosphoprotein
	8q24.22	*WISP1*	Belongs to connective tissue growth factor family		
	8q24.3	*PTP4A3*	Regulates cellular processes		
**Loss**	8p21.3–			*TNFRSF10B*	Transduces apoptosis signal
	8p23.1	*SOX7*	Potential transcriptional regulator involved in tumorigenesis		
	8p22	*CNOT7*	Binds to antiproliferative protein		
	8p22	*FGF20*	Involved in cell growth		

**Table 5.** Copy number gain and loss of genes related to cell proliferation and apoptosis in tumor DNA related to tumor progression (Dukes C + D).

**Copy number Change**	**Cytoband**	**Proliferation**	**Protein function**	**Apoptosis**	**Protein function**
**Gain**	7p22.3	*FTSJ2*	Involved in cell cycle control		
	7p15.2	*HOXA1/3*	May regulate gene expression and differentiation		
	7p14.2			*ELMO1*	May function in apoptosis and in cell migration
	7p14.1	*SFRP4*	Regulates cell growth and differentiation		
	7p14.1	*CDC2L5*	Regulate cell cycle	*CDC2L5*	Involved in apoptosis
	7p11.2	*EGFR*	Involved in control of cell growth and differentiation		
	7q11.23	*GTF2IRD1*	May be involved in cell cycle progression		
	7q11.23	*PTPN12*	Regulate cell growth, differentiation etc		
	19p13.3	*HMG20B*	Required for progression through G2 phase and entry into mitosis		
	19p13.2	*ARHGEF18*	Involved in gene transcription and cell growth		
	19p13.2	*TSPAN16*	Involved in regulation of cell growth activation, development and motility		
	19p13.2	*PPAN*	May have a role in cell growth		
	19p13.2	*EDG5*	Participate in cell proliferation	*EDG5*	Suppress apoptosis when expressed in rat HTC4 hepatoma cells
	19p13.2	*APG4*	Proposed to play a role in unregulated cell growth linked to cancer		
	19p13.13	*PRDX2*	May have a proliferative effect and play a role in cancer development		
	19p13.12	*NOTCH3*	Affects implementation of differentiation and proliferation (by similarity)	*NOTCH3*	Affects implementation of apoptosis (by similarity)
	19p13.11	*GDF15*	Regulate differentiation and maintenance		
	19p13.11			*JUND*	Protect cells from p53-dependent apoptosis and senescence
	20p13	*SOX12*	Potential role of differentiation and maintenance		
	20p13	*PTPRA*	Implicated in regulation of proliferation		
	20p13	*CDC25B*	Required for entry into mitosis. Has oncogenic properties		
	20p13	*ADRA1D*	Activate mitogenic responses and regulate cell growth and proliferation		
	20p12.3	*MCM8*	May have a role in control of cell proliferation		
	20p11.23	*RBBP9*	May play a role in the regulation of cell proliferation and differentiation		

**Table 6.** Unconfirmed CNP locus and corresponding genes with known function in DNA from normal colon tissue obtained from dead patients of potential importance for interactions to predict death events.

**Copy Number Change**	**Cytoband**	**Gene Name**	**Protein function**	**BAC clone**
**Gain**	18q11.2	*GATA-6*	Translation factor that may be important for regulating terminal differentiation and/or proliferation	RP11-121I20–RP11-219C07
		*CTAGE1*	Cutaneous T-cell lymphoma-associated antigen	
**Loss**	18q11.2	*C18orf8*	Colorectal Cancer associated protein Mic-1	RP11-197B23–RP11-626P24
		*LAMA3*	Thought to be involved in cell adhesion and signal transduction	

**Table 7.** BAC-clones ranked by the greatest differences in log2 ratio between tumor DNA from dead and alive patients and genes of either interest^a^ or non-interest^b^ that map to corresponding regions. Gains and losses reflect the aberration in DNA from dead patients when DNA from alive patients lacks the alteration or shows converted properties.

**Copy Number Change**	**Cytoband**	**BAC clone**	**Gene Name**	**Protein function**
**Gain**	8q23.3	RP11-771F4	*b*	
	8q24.3	RP11-105P9	*c*	
	10p13	RP11-609G23	*b*	
	12p12.3	CTD-2009E21	*b*	
	8q24.13	RP11-532K12	*HAS2*	Produced during wound healing and tissue repair to provide a framework for ingrowth of blood vessels and fibroblasts
	8q24.12	RP11-389M7	*b*	
	3q25.2	RP11-597G4	*c*	
	8q24.13	RP11-293H22	*b*	
	4q13.3	RP11-393B3	*b*	
	8q24.21	RP11-739G15	*c*	
**Loss**	1q23.1	RP11-769J1	*CD1*	T-cell surface glycoprotein, associates non-covalently with beta-2-microglobulin
	5q31.3	RP11-614D16	*b*	
	11q12.2	RP11-565O16	*PRPF19 CD8*	Implicated in double-strand break repair T-cell differentiation antigen
	4q12	RP11-777P23	*b*	
	12q21.33	RP11-632B21	*c*	
	17q11.2	RP11-518B17	*NF1*	Negative regulator of the ras signal transduction pathway
	12q14.1	CTD-2260D13	*b*	
	10q22.3	RP11-732B12	*c*	
	11q14.3	RP11-715F05	*b*	
	22q11.21	RP1154C2	*b*	

*^a^*Gene related to cell proliferation, apoptosis or cell cycle associated proteins.

*^b^*Genes without obvious interest for tumor progression.

*^c^*No reported genes map to the region covered by this BAC-clone.
